# The effect of total arterial grafting on medium-term outcomes following coronary artery bypass grafting

**DOI:** 10.1186/1749-8090-2-44

**Published:** 2007-10-23

**Authors:** Jean-Francois Légaré, Ansar Hassan, Karen J Buth, John A Sullivan

**Affiliations:** 1 Queen Elizabeth II Health Sciences Center, Halifax, Nova Scotia, Canada

## Abstract

**Background:**

While it is believed that total arterial grafting (TAG) for coronary artery bypass grafting (CABG) confers improved long-term outcomes when compared to conventional grafting with left internal mammary artery and saphenous vein grafts (LIMA+SVG), to date, this has not become the standard of care. In this study, we assessed the impact of TAG on medium-term outcomes after CABG.

**Methods:**

Peri-operative data was prospectively collected on consecutive first-time, isolated CABG patients between 1995 and 2005. Patients were divided into two groups based on grafting strategy: TAG (all arterial grafts no saphenous veins) or LIMA+SVG. Patients who had an emergent status or underwent fewer than two distal bypasses were excluded. Medium term univariate and risk-adjusted comparisons between TAG and LIMA+SVG cases were performed.

**Results:**

A total of 4696 CABG patients were included with 1019 patients undergoing TAG (22%). Unadjusted in-hospital mortality was 1.5% for TAG patients compared to 2.0% for LIMA+SVG (p = 0.31). The mean follow-up was 4.8 ± 2.0 years for TAG patients compared to 6.1 ± 3.0 years for LIMA+SVG patients (p < 0.0001). At follow-up total mortality (8% vs 19%; p < 0.0001), and the incidence of readmission to hospital for cardiac reasons (29% vs 38%; p < 0.0001) were significantly lower in TAG compared to LIMA+SVG patients. However, after adjusting for clinical covariates, TAG did not emerge as a significant independent predictor of long-term mortality (HR 0.92; CI 0.71–1.18), readmission to hospital (HR 1.02; CI 0.89–1.18) or the composite outcome of mortality and readmission (HR 1.00; CI 0.88–1.15). Risk adjusted survival was better than 88% in both TAG and LIMA-SVG patients at 5 years follow-up.

**Conclusion:**

Patients undergoing TAG appear to experience lower rates of medium-term all-cause mortality and readmission to hospital for any cardiac cause when compared to patients undergoing LIMA+SVG. However, after adjusting for clinical variables, this difference no longer persists suggesting that at median follow-up there are no mortality or morbidity benefit based on the choice of conduit.

## Introduction

Since its introduction nearly four decades ago, coronary artery bypass grafting (CABG) has established itself as an important therapeutic intervention for patients with symptomatic coronary artery disease. In addition to providing symptom relief, CABG has been shown to provide significant survival benefits with acceptable post-operative morbidity and mortality. Even as the average level of co-morbid illness and surgical acuity continues to rise among patients referred for CABG, risk-adjusted rates of in-hospital mortality following CABG have steadily declined, owing in large part to the ongoing advancements in the peri-operative management of patients undergoing CABG and improved surgical technique [1,2]. While short-term results following CABG are generally undisputed, long-term results have been more uncertain.

During the early to mid 1980's, reports emerged in the literature describing the considerably diminished long-term patency rates of saphenous vein grafts (SVG), eventually leading to SVG occlusion which would result in the inevitable recurrence of symptoms, the need for readmission to hospitalization and coronary re-intervention and possibly even death [3,4]. However, around the same time, publications exalting the superior long-term patency rates of the left internal mammary artery (LIMA) graft and the improved long-term survival associated with grafting of the LIMA to the left anterior descending (LAD) artery surfaced, re-establishing confidence in the long-term benefits of CABG [3-5]. Consequently, LIMA used in combination with SVG (LIMA+SVG) became the standard or conventional strategy employed during CABG procedures.

With the success of LIMA grafts, it seemed intuitive that using other arterial conduits such as the right internal mammary artery (RIMA), the radial artery (RA) and/or the gastroepiploic artery (GEPA) in conjunction with LIMA grafts would further improve long-term outcomes. In fact, studies showed that LIMA + RIMA was associated with improved long-term survival and diminished rates of angina recurrence and late MI and that RA grafts were associated with improved short-term and long-term patency rates [6-10]. These results generated considerable interest in the complete elimination of SVG altogether and the institution of total arterial grafting (TAG) for the purposes of improving long-term results following CABG [11-20].

To date, studies looking at the efficacy of TAG as compared to traditional strategies using LIMA+SVG have focused primarily on short-term outcomes following surgery and have provided mixed results. Royse et al demonstrated that TAG was associated with reduced in-hospital mortality, while Muneretto et al, in a series of randomized controlled trials, showed that while rates of in-hospital mortality were not different between the two groups, incidence of angina recurrence and graft occlusion at approximately one year was significantly lower in the group receiving TAG [16-19]. Legare et al, using propensity score analysis, compared patients undergoing TAG with composite arterial grafts to a matched group of patients receiving LIMA+SVG and found that TAG was independently associated with higher rates of post-operative morbidity even though a mortality difference was not demonstrable [20]. More recently, Guru et al reported improved risk-adjusted survival and greater freedom from cardiac morbidity in patients with multiple arterial grafts (12% of patients with multiple arterial grafts) as compared to hose with single arterial grafts [21].

The objective of this study is to determine the effect of TAG on medium-term outcomes following CABG. This study addresses some of the limitations of previous studies in terms of sample size, duration of follow-up and confounding introduced by the inclusion of patients with multiple but not total arterial grafts.

## Materials and methods

### Patients

All patients who underwent isolated, on-pump CABG at the Queen Elizabeth II (QEII) Health Sciences Center from 1995 until 2003, in Halifax, Canada, were identified using the Maritime Heart Center (MHC) database. From this group, only those patients who underwent CABG using TAG or LIMA+SVG were included for consideration in the final analysis. Patients were excluded from the final analysis if they underwent single-vessel bypass surgery or if they required an emergent or emergent salvage procedure. In both groups only patients with a LIMA to the left anterior descending artery were included.

### Operative Technique

All interventions were performed via a midline sternotomy, and cardiopulmonary bypass was instituted in a standardized manner for all cases. Briefly, body temperature during the procedure was allowed to drift to 32°C. Intermittent cold cardioplegia solution was delivered antegrade via the aortic root unless otherwise indicated. Arterial conduits were harvested with minimal trauma (LIMA and RIMA were not skeletonized) and were treated with either a papaverine solution or a nitroglycerine/calcium channel blocker (verapamil) solution prior to their use.

TAG was defined as the use of any arterial conduit (LIMA, RIMA, RA or GEPA), either alone or in combination, without concomitant use of SVG. The choice of conduit and the manner in which the grafts were constructed, including whether or not grafts were constructed in a composite T- or Y- fashion, proximal aorto-coronary anastomoses were performed or sequential anastomoses were used, was based entirely on surgeon preference rather than on any fixed criteria such as territory to graft or degree of target vessel stenosis. LIMA+SVG, on the other hand, was defined as any case in which the LIMA was used for the purposes of a single bypass to the left anterior coronary artery (LAD) and the remaining bypasses were carried out using SVG. In these cases, SVG were constructed as either a series of sequential anastomoses or as single bypasses and were anastomosed proximally to the aorta.

### Post-operative Management

All patients received intravenous nitroglycerine infusions for the first 24 hours upon return from the operating room unless hypotensive (systolic blood pressure < 90 mm Hg). Oral nifedipine (Adalat 10 mg by mouth 4 times a day or Adalat extended release (XL) 20 to 30 mg by mouth daily; Bayer Inc., Toronto, Ontario, Canada) was prescribed for all patients receiving a radial artery beginning on day 1 post-operatively for a period of 3 to 6 months. Other routine post-operative medications included daily aspirin as well as resumption of cholesterol lowering agents, β-blockers and angiotensin converting enzyme inhibitors as appropriate.

### Data Sources

The MHC database captures detailed information on a wide range of pre-operative, intra-operative, and in-hospital post-operative variables including post-operative complications and in-hospital mortality for all patients undergoing cardiac surgery at the QEII Health Sciences Center in Nova Scotia, Canada. In order to gather information regarding long-term outcomes, the MHC database was linked to the Canadian Institute for Health Information (CIHI) Discharge Abstract Database and the Nova Scotia Vital Statistics database. The CIHI Discharge Abstract Database is a national database that contains extensive data for each inpatient and outpatient hospital visit in Nova Scotia and enables us to track all readmissions to hospital. The Nova Scotia Vital Statistics database collects information on all births and deaths occurring within the province of Nova Scotia.

### Variable Selection

Pre-operative variables of interest included age (age ≥ 70 vs. age < 70), gender, body mass index or BMI (BMI > 25 kg/m^2 ^vs. BMI = 25 kg/m^2^), smoking history, diabetes, hypercholesterolemia, renal insufficiency (pre-operative serum creatinine of ≥ 176 μmol/L), hypertension, peripheral and/or cerebrovascular disease, left ventricular ejection fraction or EF (EF < 40% vs. EF ≥ 40%), recent myocardial infarction (MI) defined as the occurrence of an MI in the 21 days prior to surgery, pre-operative inotropes/intra-aortic balloon pump (IABP) use, New York Heart Association (NYHA) functional classification (NYHA class IV vs. NYHA classes I – III), urgency status (operation performed within 24 hours from the time of referral vs. operation performed at a time interval of greater than 24 hours from the time of referral), prior percutaneous coronary intervention (PCI) and number of diseased vessels (triple-vessel or left main disease vs. single- or double-vessel disease). As mentioned earlier, patients undergoing emergent or emergent salvage procedures were excluded. Intra-operative variables of interest included number of distal anastomoses, cross clamp time and total bypass time. The medium-term outcomes of interest included all-cause mortality following discharge from hospital, readmission to hospital for any cardiac cause as defined by the following codes from the ninth revision of the International Classification of Disease, Clinical Modification (ICD-9-CM) (20): 410 (acute myocardial infarction), 411 (unstable angina), 412 (old myocardial infarction), 413 (angina pectoris), 414 (other forms of chronic IHD), 426 (conduction disorders), 427 (cardiac dysrhythmias), 428 (heart failure), 429 (ill-defined descriptions and complications of heart disease), coronary re-intervention (PCI or CABG) and a composite outcome defined as all-cause mortality following discharge from hospital or readmission to hospital for any cardiac cause.

### Statistical Analysis

Univariate comparisons between cases performed using TAG and cases performed using LIMA+SVG were carried out based on pre-, intra-, and post-operative variables including rates of in-hospital mortality and long-term adverse events using Chi-square tests for dichotomous variables and two-tailed t-tests for continuous variables. Unadjusted rates of the long-term composite outcome were then compared between TAG and LIMA+SVG cases using Kaplan Meier survival plots and the log-rank test. A fully-adjusted Cox proportional hazard model was created to determine the effect of TAG on the long-term outcome of interest after adjusting for differences between patients in clinical presentation. Pre-operative variables regardless of whether or not they differed between TAG and LIMA+SVG were included in the model building process, and a non-parsimonious model was created that retained all variables. The following variables were used in the model: age groups, COPD, gender, diabetes, preop renal insufficiency, cerebrovascular disease, peripheral vascular disease, EF groups, congestive heart failure, urgency status, number of diseased vessels, group assignment (TAG vs LIMA+SVG) and propensity analysis score (p score). A propensity score was calculated for each patient using the predicted probability of being in the TAG group. A multinomial logistic regression model was used to predict the probability of TAG group assignment (p score) after adjusting for all relevant preoperative patient characteristics including era (1995–2000 vs 2000–2005). Statistical significance was set at p < 0.05. All statistical analyses were performed using the SAS software package version 8.2 (SAS, Cary, North Carolina).

## Results

A total of 4696 patients underwent first-time, isolated, on-pump, non-emergent CABG with greater than a single bypass between March 1, 1995 and March 31, 2005 using either TAG (n = 1019) or LIMA+SVG (n = 3677). Patient characteristics of the two groups are shown in Table 1. When compared to patients undergoing LIMA+SVG, patients receiving TAG were younger, more likely to be male and less likely to have hypercholesterolemia, renal failure, peripheral vascular disease and triple vessel or left main disease. TAG patients also had better heart function and were operated more on an elective basis.

**Table 1:** Comparing pre- and intra-operative characteristics among patients undergoing CABG with TAG and with LIMA+SVG.

**Variable**		**TAG n (%)**	**LIMA+SVG n (%)**	**p-value**
**Age**		60.2 ± 9.7 yrs	65.8 ± 9.8 yrs	< 0.0001
**Female sex**		205 (20)	995 (27)	< 0.0001
**Smoking history**		732 (72)	2565 (70)	0.20
**Diabetes**		347 (34)	1360 (37)	0.08
**Hypercholesterolemia**		902 (88)	2703 (74)	< 0.0001
**Pre-op renal failure**		16 (2)	218 (6)	< 0.0001
**Hypertension**		629 (62)	2368 (64)	0.12
**Peripheral vascular disease**		138 (14)	623 (17)	0.009
**Cerebrovascular disease**		84 (8)	525 (14)	< 0.0001
**Ejection fraction**	**> 50**	831 (82)	2711 (76)	< 0.0001
	**30–50**	168 (16)	706 (19)	
	**< 30**	20 (2)	197 (5)	
**Recent MI < 7d**		36 (4)	208 (6)	0.007
**Pre-op IABP**		27 (3)	182 (5)	0.002
**CHF**		77 (8)	539 (15)	< 0.0001
**COPD**		120 (12)	429 (14)	0.033
**Urgent (< 24 hours)**	**Elective**	615 (60)	1811 (49)	< 0.0001
	**IHU**	331 (32)	1421 (39)	
	**Urgent**	73 (7)	445 (12)	
**Prior PCI**		126 (12)	366 (10)	0.026
**Left-main/triple-vessel disease**		789 (77)	3145 (86)	< 0.0001
**Number of bypasses**		3.1+0.9 grafts	3.2+0.8 grafts	< 0.0001

BMI, Body mass index; CHF, history of congestive heart failure; COPD, chronic obstructive lung disease; MI, myocardial infarction; IABP, intra-aortic balloon pump; IHU, in-hospital urgent i.e. waiting for surgery in-hospital longer than 24 hrs; PCI, percutaneous coronary intervention.

The proportion of TAG was 22% of all CABG patients during the study period. The conduits used for TAG patients were: the left internal mammary artery (LIMA; 100%), right internal mammary artery (RIMA; 39%), and radial artery (62%). Overall there were fewer distal anatomoses performed in TAG patients (3.1 ± 0.9 grafts) compared to LIMA+SVG patients (3.2 ± 0.8 grafts; p < 0.001) (Table 1). Unadjusted in-hospital mortality was 1.5% for TAG patients compared to 2.0% for LIMA+SVG (p = 0.31). Similarly unadjusted rates of in-hospital permanent stroke or post-operative myocardial infarction did not differ significantly between the two groups (Table 2). The unadjusted median length of hospitalization was 6 days (IQR 5–7) for TAG group compared to 7 days (IQR 5–8) for the LIMA+SVG group (p < 0.0001).

**Table 2:** In-hospital and long-term outcomes following CABG with TAG and with LIMA+SVG

**Outcome**	**TAG (%)**	**LIMA+SVG (%)**	**p-value**
In-hospital outcomes			
Mortality	1.5	2.0	0.31
Stroke	1.6	1.8	0.67
Myocardial infarction	1.2	1.1	0.87
			
Long-term outcomes			
All-cause mortality	8.4	18.6	< 0.0001
Readmission for any cardiac cause	28.8	37.9	< 0.0001
Composite outcome	33.5	46.6	< 0.0001

**Figure 1:**
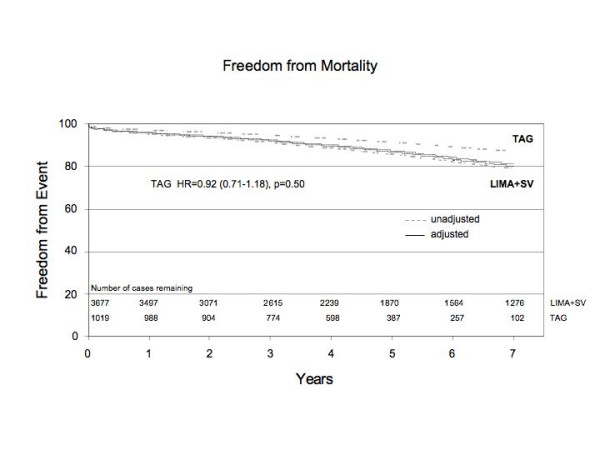
Risk adjusted freedom from mortality between TAG group and LIMA+SVG group.

The median follow-up was 4.5 ± 2.0 years for TAG patients compared to 6.2 ± 3.0 years for LIMA+SVG patients (p < 0.0001). Over the entire follow-up period, 86 patients (8.4%) had died in the TAG group compared to 683 patients (18.6%) in the LIMA+SVG group (p < 0.0001) (Table 2). After adjusting for clinical covariates, TAG did not emerge as a significant independent predictor of long-term mortality (HR 0.92; CI 0.71–1.18). Risk adjusted survival was better than 88% in both TAG and LIMA-SVG patients at 5 years follow-up (Figure 1).

**Figure 2:**
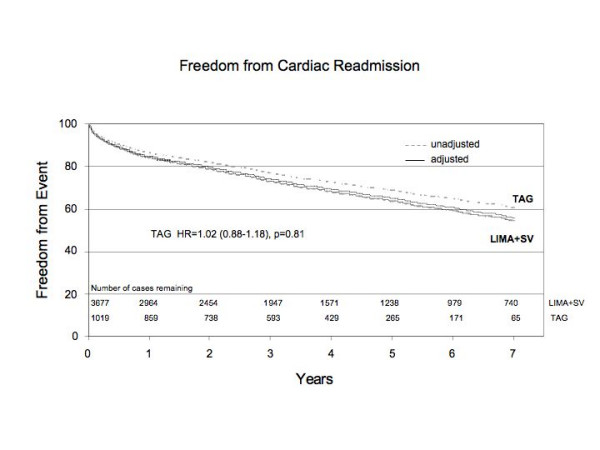
Risk adjusted freedom from readmission to hospital for cardiac reason between TAG group and LIMA+SVG group.

Following discharge 29% of TAG patients were readmitted to hospital for any cardiac reasons as compared to 38% of LIMA+SVG patients (p < 0.0001). This included 58 (5.7%) TAG patients vs 153 (4.3%) LIMA+SVG patients who were readmitted for repeat revascularization in the form of PCI or CABG surgery (p = 0.04). The incidence of repeat CABG was 5 (0.5%) TAG patients compared to 31 (0.8%) LIMA+SVG patients (p = 0.25). After adjusting for clinical covariates, TAG did not emerge as a significant independent predictor of readmission to hospital (HR 1.02; CI 0.88–1.18). Risk adjusted freedom from readmission to hospital, for any cardiac reason curves are illustrated in figure 2.

**Figure 3:**
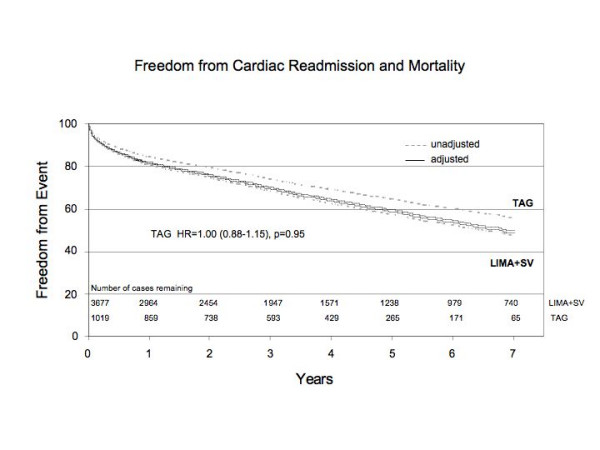
Risk adjusted freedom from the composite outcome: death and readmission to hospital for cardiac reason TAG group and LIMA+SVG group.

We then used the composite outcome of death or readmission to hospital for cardiac reason to compare TAG and LIMA+SVG patients. Utilizing unadjusted data we were able to show that TAG offers a significantly higher freedom from the composite adverse outcome when compared to LIMA+SVG patients (Figure 3). However, after adjusting for clinical covariates, TAG was no longer associated with improved freedom from the composite outcome of all-cause mortality or readmission to hospital for any cardiac cause over time (HR 1.00, 95% CI 0.88 – 1.15) (Figure 3).

The following variables were found to be independent predictors of the composite outcome (death and/or readmission to hospital for any cardiac cause): increased age (> 70), COPD, female gender, diabetes, pre-operative renal insufficiency, peripheral vascular disease, low ejection fraction (< 50%), history of congestive heart failure and patient requiring surgery while hospitalized (Table 3).

**Table 3:** Fully-adjusted Cox-proportional hazards model with long-term composite outcome as the outcome (Death and/or readmission to hospital for cardiac reason)

**Variable**	**HR**	**95% CI**	**p-value**
Age 70–79	1.25	1.10–1.41	0.0004
Age 80+	1.55	1.25–1.88	< 0.0001
COPD	1.39	1.24–1.57	< 0.0001
Female gender	1.14	1.04–1.26	0.006
Diabetes	1.28	1.17–1.40	< 0.0001
Pre-op renal failure	1.75	1.48–2.07	< 0.0001
Peripheral vascular disease	1.40	1.25–1.57	< 0.0001
Ejection fraction 30–49	1.22	1.09–1.36	0.006
Ejection fraction < 30	1.38	1.13–1.67	0.001
Congestive heart failure	1.33	1.17–1.52	< 0.0001
In-hospital urgent IHU (> 24 hours)	1.38	1.25–1.52	< 0.0001
Urgent (< 24 hours)	1.50	1.31–1.72	< 0.0001
*pscore*	*0.86*	*0.67–1.10*	*0.222*

*Total arterial grafting (TAG)*	*0.92*	*0.76–1.11*	*0.397*

## Discussion

The poor long-term patency rates of SVG coupled with reports citing the excellent long-term patency rates of and improved survival associated with arterial grafts have encouraged many to abandon the use of SVG in favour of revascularization performed exclusively with arterial conduits[4-6,8,10]. The apparent increasing implementation of TAG has been greatly facilitated by the development of innovative surgical techniques, including Y- and T- graft constructions, skeletonization of the internal mammary arteries, as well as by the resurgence of sequential anastomoses [12-14]. While many case series have commented on the safety of TAG, few studies have actually compared outcomes following TAG with outcomes following the traditional grafting strategy of LIMA+SVG [22]. Those that have compared the two grafting strategies have reported mixed short-term results (16 – 20), and data describing long-term outcomes, where one would expect to see the greatest impact of TAG, is lacking [16-19]. Taken together TAG does not appear to have gained wide acceptance as the standard of care which is best exemplified by the relative low proportion (~10%) of CABG patients receiving exclusive arterial grafts[21,22].

In this study, we found that patients undergoing CABG with TAG had fewer co-morbid illnesses and were more likely to present with diminished coronary disease severity. In-hospital mortality did not differ significantly between the two groups, but medium-term survival and freedom from readmission to hospital was significantly improved in patients undergoing TAG. However, when differences between the two groups in terms of age, gender, co-morbid illness, coronary disease severity and surgical acuity were adjusted for using Cox proportional hazard modeling techniques, TAG no longer emerged as an independent predictor of freedom from long-term adverse events following CABG. Unlike Lytle et al we were unable to show that increased extent of arterial grafting performed at primary coronary artery bypass grafting decreased occurrence of coronary reoperation [23].

This retrospective study provides important comparisons of medium-term outcomes between patients undergoing CABG with TAG and patients undergoing CABG with the more traditional LIMA+SVG. The absence in this study of any impact of TAG on risk-adjusted long-term rates of all-cause mortality and readmission to hospital for any cardiac cause reaffirms the safety of this grafting strategy. To date, retrospective studies comparing TAG and LIMA+SVG have focused on in-hospital outcomes [16,20]. The only studies that have compared TAG with LIMA+SVG on the basis of both in-hospital and long-term outcomes were a series of randomized clinical trials by Muneretto et al that showed that while in-hospital morbidity and mortality were similar between the two groups, TAG was associated with increased freedom from the combined adverse outcome of non-fatal MI, angina recurrence, graft occlusion, need of PCI re-intervention and late death among patients over the age of 50 as well as among elderly patients over the age of 70 [17-19]. It should be noted, though, that these trials were limited by reduced sample sizes (n = 200) and limited duration of follow-up (mean follow-up 12 to 16 months). In addition, the manner in which TAG was performed was restricted exclusively to composite graft construction, which further limits the generalizability of their results. More recently a large retrospective study from Guru et al suggested that there is both a survival and morbidity benefit to multiple arterial grafts as compared to single arterial grafts [21]. The major strengths of their study were the large size and length of follow-up. However, their results are also difficult to generalize as they were derived from fewer than 6% of patients with 3-vessel coronary disease and a relative small proportion of selected patients receiving multiple arterial grafts (~12% of CABG patients).

This study is not without its limitations, it can be argued that the length of follow-up in this study is insufficient to discern whether long-term rates of adverse events in fact differ between patients undergoing CABG with TAG and patients undergoing CABG with LIMA+SVG. The average length of follow-up for this study for patients undergoing TAG was 4.5 years, which is longer than the length of follow-up from many previously published retrospective or prospective analyses that have attempted to compare TAG with conventional CABG. However, despite this limitation we provide data on a large cohort (n = 1053) of exclusively TAG patients compared to conventional LIMA+SVG something very few have done before and demonstrate the safety of TAG with the potential for long term benefit. Secondly, because of the many different ways in which TAG can be accomplished surgically, a simple comparison between all cases of CABG with TAG and cases of CABG with LIMA+SVG underestimates, to some extent, the complexity of the various TAG arrangements and the differing impact that each arrangement may have on long-term rates of adverse outcomes. For example, by including cases in which composite Y- or T- grafts were used together with cases in which a free arterial conduit was anastomosed proximally to the aorta, we may be ignoring the important effect that composite grafts may have on post-operative outcomes independent of the whether or not the CABG was performed with TAG. However, despite of the underlying heterogeneity in surgical technique and level of difficulty found among the different TAG arrangements, we felt that the inclusion of the various arrangements under a single entity more accurately represented a typical practice in which TAG was performed on a regular basis and better answered the broader question of whether CABG performed with TAG was superior to CABG performed with LIMA+SVG.

In conclusion, when compared to patients undergoing CABG with LIMA+SVG, patients presenting for CABG with TAG did so with fewer comorbidities and less coronary disease severity. While in-hospital mortality rates were similar between the two groups, patients undergoing CABG with TAG had significantly lower medium-term rates of all-cause mortality and readmission to hospital for any cardiac cause. Following adjustment for differences between patients in terms of age, co-morbid illness and extent of coronary disease, TAG did not emerge as a significant predictor of freedom from medium-term adverse events following CABG.

## Abbreviations

CABG Coronary artery bypass surgery

CI Confidence interval

GEPA Gastroepiploic artery

HR Hazard ration

IQR Interquartile range

LIMA Left internal mammary artery

PCI Percutaneous coronary intervention

RA Radial artery

RIMA Right internal mammary artery

SVG Saphenous vein graft

TAG Total arterial grafting

## Authors' contributions

All authors have read and approved the final manuscript.

JFL Conceived the present study, participated in the analysis and drafting of the manuscript

AH Participated in the design, analysis and drafting of the manuscript

KJB Was instrumental to the statistical analysis and critical appraisal of the manuscript

JAS Participated in the analysis and drafting of the manuscript
